# Integrated metabolomic and transcriptomic analyses of regulatory mechanisms associated with uniconazole-induced dwarfism in banana

**DOI:** 10.1186/s12870-022-04005-w

**Published:** 2022-12-28

**Authors:** Liuyan Qin, Chaosheng Li, Chenglin Guo, Liping Wei, Dandan Tian, Baoshen Li, Di Wei, Wei Zhou, Shengfeng Long, Zhangfei He, Sumei Huang, Shaolong Wei

**Affiliations:** 1 Biotechnology Research Institute, Guangxi Academy of Agricultural Sciences, Nanning, 530007 China; 2 Biotechnology Research Institute, Guangxi Academy of Agricultural Sciences/National Local Joint Engineering Research Center for Genetic Improvement and Cultivation Techniques of Banana Varieties/National Tropical Fruit Variety improvement Center Guangxi Banana Branch Center, Nanning, 530007 China; 3 Institute of Plant Protection, Guangxi Academy of Agricultural Sciences, Nanning, 530007 China

**Keywords:** Banana dwarfing, Uniconazole, Metabolome, Transcriptome, Flavonoid synthesis

## Abstract

**Background:**

Uniconazole is an effective plant growth regulator that can be used in banana cultivation to promote dwarfing and enhance lodging resistance. However, the mechanisms underlying banana dwarfing induced by uniconazole are unknown. In uniconazole-treated bananas, gibberellin (GA) was downregulated compared to the control groups. An integrative analysis of transcriptomes and metabolomes was performed on dwarf bananas induced by uniconazole and control groups. The key pathways involved in uniconazole-induced dwarfism in banana were determined according to the overlap of KEGG annotation of differentially expressed genes and (DEGs) differential abundant metabolites (DAMs).

**Results:**

Compared with the control groups, the levels of some flavonoids, tannins, and alkaloids increased, and those of most lipids, amino acids and derivatives, organic acids, nucleotides and derivatives, and terpenoids decreased in uniconazole-treated bananas. Metabolome analysis revealed the significant changes of flavonoids in uniconazole-treated bananas compared to control samples at both 15 days and 25 days post treatment. Transcriptome analysis shows that the DEGs between the treatment and control groups were related to a series of metabolic pathways, including lignin biosynthesis, phenylpropanoid metabolism, and peroxidase activity. Comprehensive analysis of the key pathways of co-enrichment of DEGs and DAMs from 15 d to 25 d after uniconazole treatment shows that flavonoid biosynthesis was upregulated.

**Conclusions:**

In addition to the decrease in GA, the increase in tannin procyanidin B1 may contribute to dwarfing of banana plants by inhibiting the activity of GA. The increased of flavonoid biosynthesis and the change of lignin biosynthesis may lead to dwarfing phenotype of banana plants. This study expands our understanding of the mechanisms underlying uniconazole-induced banana dwarfing.

**Supplementary Information:**

The online version contains supplementary material available at 10.1186/s12870-022-04005-w.

## Background

Dwarfing, an important agronomic trait, has been a research hotspot for breeders. Varieties with dwarfing characteristics not only have unique advantages in production management but also have great potential in terms of high yield. In the production of bananas (*Musa* spp.), the stems of tall bananas are easily damaged by typhoons and require additional support costs. Production efficiency can be optimized by controlling grass growth in bananas. Semi-dwarf banana varieties are resistant to wind and rain damage [1]. In addition, an increase in yield associated with short stems is associated with an increase in the harvest index [2]. The crouching physique of dwarf banana varieties can resist the harm of typhoons to a certain extent and has the advantages of convenient cultivation, field management, labor saving, and dense planting [3]. The dwarf variant is also helpful for mining and researching dwarf-related genes. The identification and utilization of dwarf-related banana genes are very important for breeding dwarf banana varieties.

Plant height traits are not only controlled by internal genes, but are also affected by various hormones and external environmental factors [4]. Studies have found that most dwarf mutations are caused by changes in the growth and development of plant stems owing to mutations in hormone synthesis pathways or response regulation [5]. Many dwarf mutants are related to plant gibberellins (GAs) and brassinosteroids, whereas several mutations are related to auxins [6, 7]. By analyzing plant hormone synthesis and signaling mutants and treating plants with exogenous hormone spray, it was found that GAs and brassinosteroids regulate the expansion of plant cells and organs along the longitudinal axis, greatly affecting plant height and organ size [8]. They are the main hormones that cause plant dwarfing. Uniconazole, a highly effective plant growth inhibitor, hinders the oxidative demethylation of kaurene to kaurenoic acid, making it difficult to synthesize kaurenoic acid and thereby cutting off the biosynthesis of GAs [9]. At the same time, it is an effective dwarf plant inducer [10]. At present, the application of uniconazole in fruits is mainly used to control vegetative growth, promote plant rooting and flower formation, promote seed filling, improve fruit quality, increase yield, and enhance stress resistance [11, 12]. Previous studies on the dwarfing mechanisms of model plants such as *Arabidopsis thaliana* and rice have been conducted, and it is believed that plant dwarfing is mainly regulated by plant hormones [4]. However, few studies have been conducted on the causes and mechanisms underlying banana dwarfing.

GAs play a fundamental role in plant growth and development and are involved in regulating a variety of developmental processes. The reduction in reactive GAs content results in plants exhibiting a dwarf phenotype. The GA biosynthetic pathway is well understood in model plants and related variants have been isolated [5, 13, 14]. GAs are biosynthesized from geranyl diphosphate, which is a common C20 precursor of diterpenoids. GA20ox, GA3ox, and GA2ox are enzymes that catalyze late reactions in the GA biosynthetic pathway and belong to the 2OG-Fe (II) oxidase superfamily. In many plant species, enzymes are independently encoded by different gene families [15] and thus have functional redundancy and tissue specificity [16]. The loss-of-function of these GA oxidase genes (except GA2ox) in plants produces a dwarf phenotype that can be restored by the application of exogenous GA [16–19]. For example, the well-known Green Revolution gene sd-1 is generated by the loss-of-function of OsGA20ox2 in rice [20]. Conversely, GA2ox reduces the levels of reactive GAs in plants, and overexpression of GA2ox leads to dwarf plants [21, 22]. Chen et al. revealed that MaGA20ox4, MaGA20ox5, MaGA20ox7, MaGA2ox7, MaGA2ox12, and MaGA2ox14 are the main genes regulating the GA content difference between 8818 and its dwarf mutant, 8818–1, and each gene may perform different functions in different tissues or during different developmental stages [1].

Banana genome sequencing was completed in 2012 [23], but related information on dwarfism metabolism in bananas is limited. The genes, pathways, and metabolites associated with dwarfism induced by uniconazole in bananas have not been explored. In this study, an integrative analysis of transcriptomes and metabolomes was performed on uniconazole-induced bananas to investigate the regulatory mechanisms of dwarfism during banana development. The results of this study provide both candidate genes and novel approaches that can be used to produce improved banana traits.

## Results

### Characterization of uniconazole-induced dwarfism in banana

**Fig. 1:**
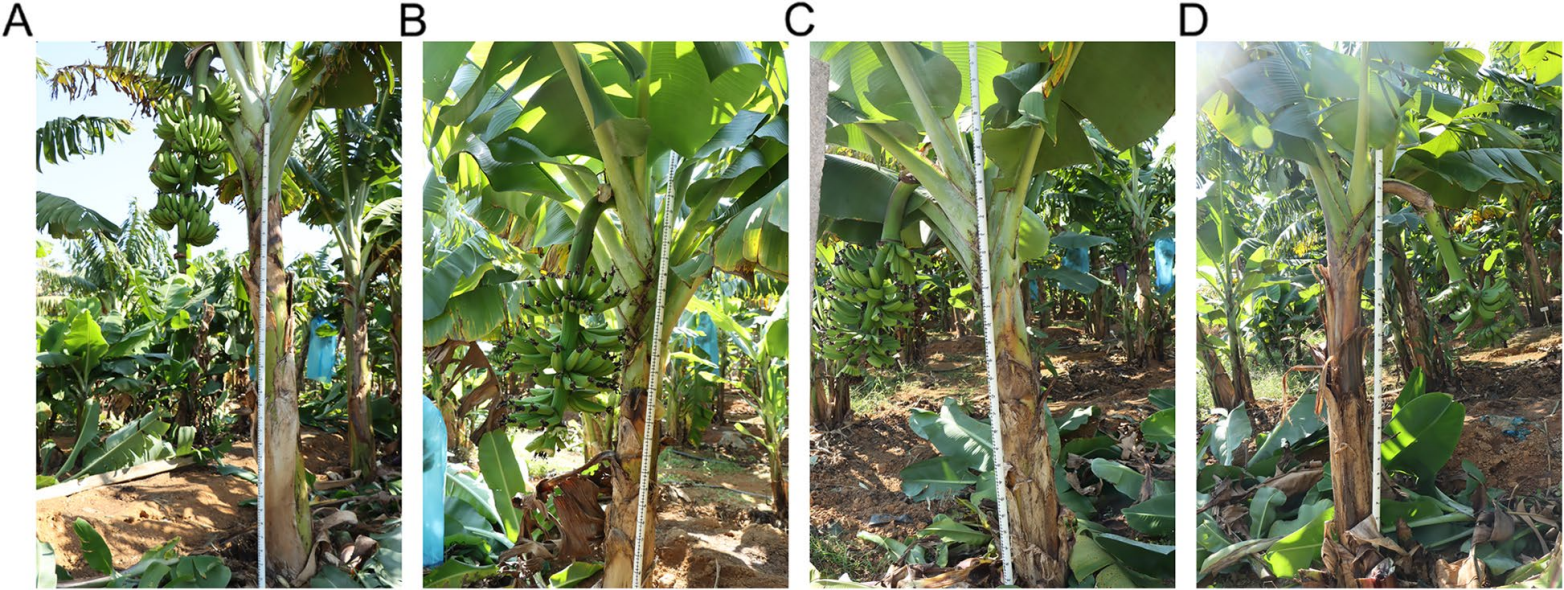
Characterization of uniconazole-induced dwarfism in banana. Plant traits were compared between control (**A**), and uniconazole treatment with a dosage of 0.1 g (**B**), 0.3 g (**C**), and 0.5 g (**D**)

To confirm the effect of uniconazole on dwarfism in bananas, we designed a concentration gradient (0.1 g, 0.3 g, and 0.5 g) and compared the induced traits (Fig. 1). The plants in the control group exhibited a height of approximately 220 cm with normal banana stalks (Fig. 1A), and plants dwarfed to approximately 170 cm when treated with 0.1 g uniconazole (Fig. 1B). Treatment with 0.3 g uniconazole further dwarfed the plants to approximately 140 cm with short comb spacing (Fig. 1C), whereas an overdose of uniconazole (0.5 g) induced malformation of buds and a height of 135 cm (Fig. 1D).

### Physiological and biochemical changes in uniconazole-induced dwarf banana

**Fig. 2:**
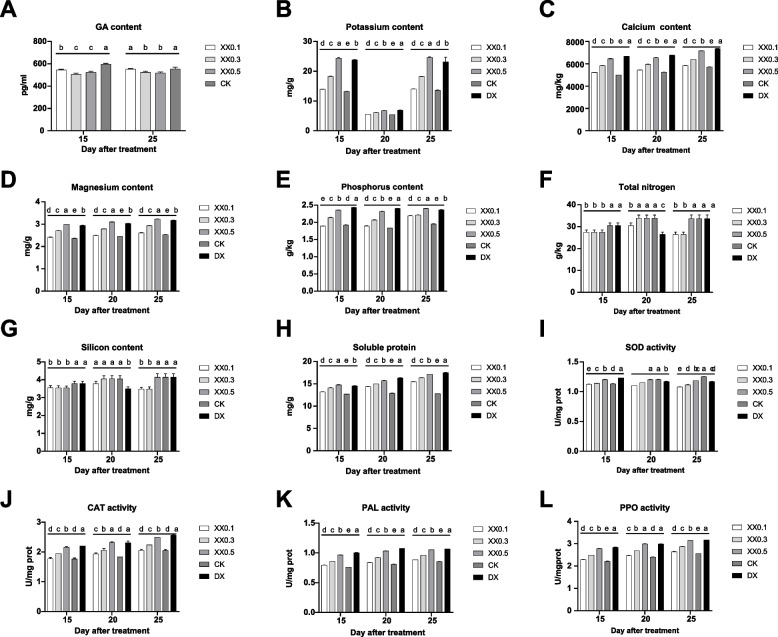
Physiological and biochemical changes induced by uniconazole treatment, including (**A**) GA, (**B**) potassium, (**C**) calcium, (**D**) magnesium, (**E**) phosphorus, (**F**) nitrogen, (**G**) silicon, (**H**) soluble protein, (**I**) SOD activity, (**J**) CAT activity, (**K**) PAL activity, and (**L**) PPO activity. XX0.1, XX0.3, and XX0.5 indicate uniconazole treatment with a dosage of 0.1 g, 0.3 g, and 0.5 g, respectively. CK and DX indicate control and treatment with paclobutrazol, respectively

GA content was significantly downregulated in uniconazole-treated bananas. The lowest GA content was observed for both groups treated with 0.3 g and 0.5 g uniconazole (Fig. 2A). A considerable number of physiological and biochemical indices, including potassium, calcium, magnesium, phosphorus, soluble protein, and SOD activity, increased as the concentration gradient of uniconazole increased. In contrast, the levels of these indices in the control and paclobutrazol-treated groups were the lowest and highest, respectively (Fig. 2B-2I). In addition, the enzyme activities of CAT, PAL, and PPO showed a similar trend (Fig. 2J-2L). Consequently, the dosage of 0.3 g uniconazole was chosen for further metabolomic and transcriptomic experiments based on to the best dwarfism traits induced in banana and the minimum effect on physiological and biochemical indices.

### Metabolomic changes associated with uniconazole-induced dwarfism

A total of 1082 metabolites were identified based on the metabolomics data (Table S1), which showed a high correlation (r > 0.9) between replicates within groups (Fig. S1A). PCA showed clustering of samples into distinct groups and stages, and the treatment group was closer to the control group at 25 d than at 15 d (Fig. S1B).

**Fig. 3:**
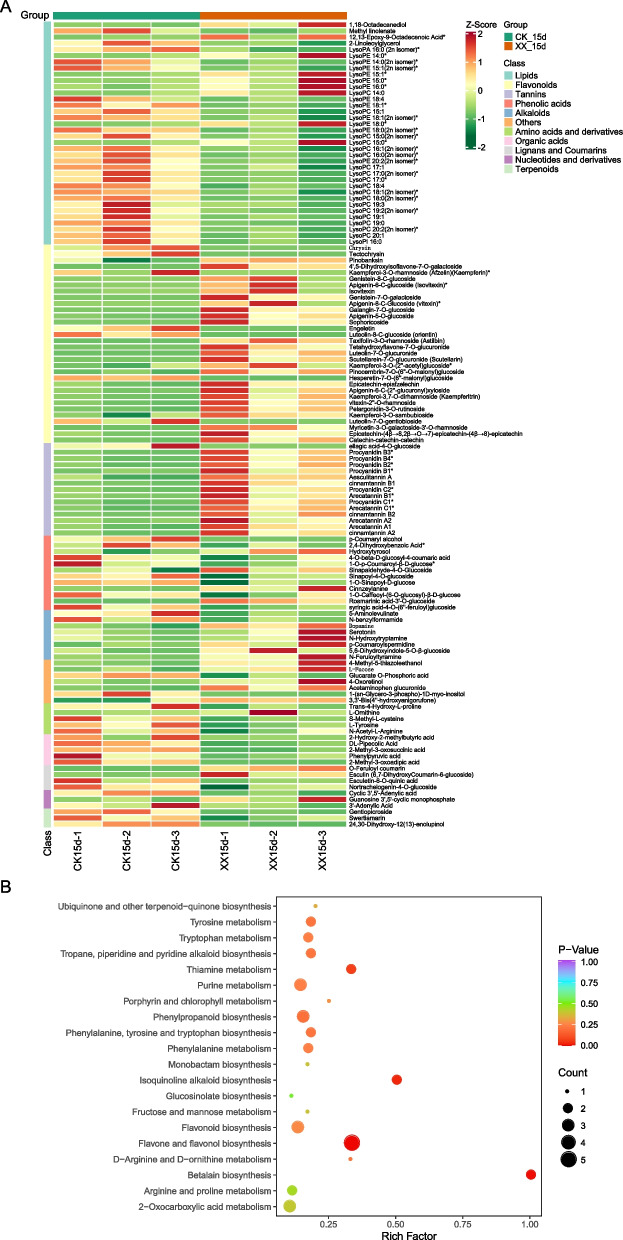
Differential metabolites (DAM) between the treatment and control groups at 15 d. **A** Heatmap representing the level of DAM across groups. **B** KEGG pathway enrichment analysis of the DAM identified in (A). XX indicate uniconazole treatment, and CK indicate control

**Fig. 4:**
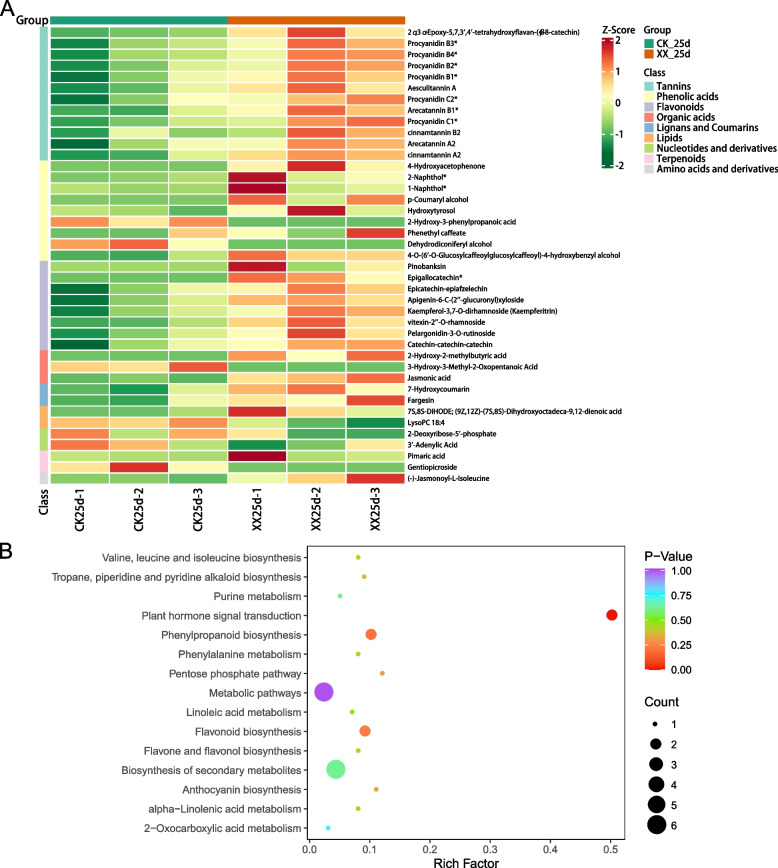
Differential metabolites (DAM) between the treatment and control groups at 25 d. **A** Heatmap representing the level of DAM across groups. **B** KEGG pathway enrichment analysis of the DAM identified in (A). XX indicate uniconazole treatment, and CK indicate control

A considerable number of differential abundant metabolites (DAMs) were identified between the treatment and control groups at 15 d (Fig. 3A). Among them, the levels of most differential flavonoids, tannins, and alkaloids increased after uniconazole induction, whereas those of most lipids, amino acids and derivatives, organic acids, nucleotides and derivatives, and terpenoids decreased (Fig. 3A). These DAMs were significantly enriched in flavone and flavonol biosynthesis, betalain biosynthesis, flavonoid biosynthesis, isoquinoline alkaloid biosynthesis, thiamine metabolism, and phenylpropanoid biosynthesis pathways (Fig. 3B)*.* Meanwhile, the levels of differential flavonoids and tannins also increased when treated with uniconazole at 25 d (Fig. 4A), and the differential metabolites participated in flavonoid biosynthesis, phenylpropanoid biosynthesis, and plant hormone signal transduction pathways (Fig. 4B). The common DAM between 15 d and 25 d showed a similar trend, including three downregulated (3′-adenylic acid, gentiopicroside, and lysoPC 18:4) and 19 upregulated metabolites, including mainly flavonoids (pinobanksin, epicatechin-epiafzelechin, apigenin-6-C-(2′-glucuronyl) xyloside, kaempferol-3,7-O-dirhamnoside (kaempferitrin), vitexin-2″-O-rhamnoside, pelargonidin-3-O-rutinoside, and catechin-catechin-catechin), and tannins (such as procyanidin, cinnamtannin, and arecatannin) (Table S2).

### Global transcriptomic changes in response to uniconazole-induced dwarfism

After the removal of low-quality reads, a minimum of 40 million clean reads were obtained for each sample and mapped to the reference genome at a high mapping rate (> 91%) (Table S3). A high correlation (r ≥ 0.87) was observed between replicates within groups for transcriptomic data (Fig. S2A), and PCA analysis shows similar clusters with the metabolomics data (Figs. S1B and S2B).

**Fig. 5:**
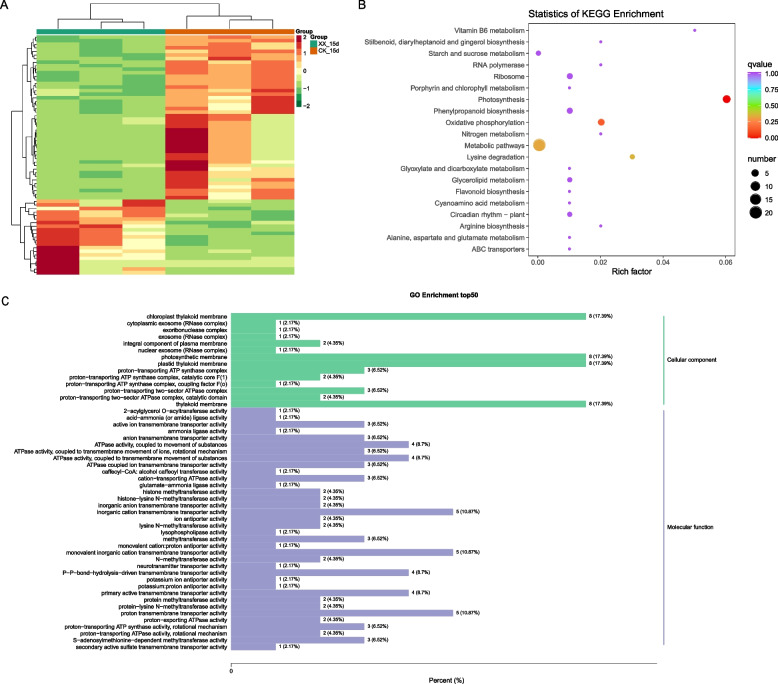
Differentially expressed genes (DEG) between the treatment and control groups at 15 d. **A** Heatmap representing the level of DEG across groups. **B** KEGG pathway enrichment analysis of the DEG identified in (A). **C** GO enrichment analysis of the DEG identified in (A). XX indicate uniconazole treatment, and CK indicate control

**Fig. 6:**
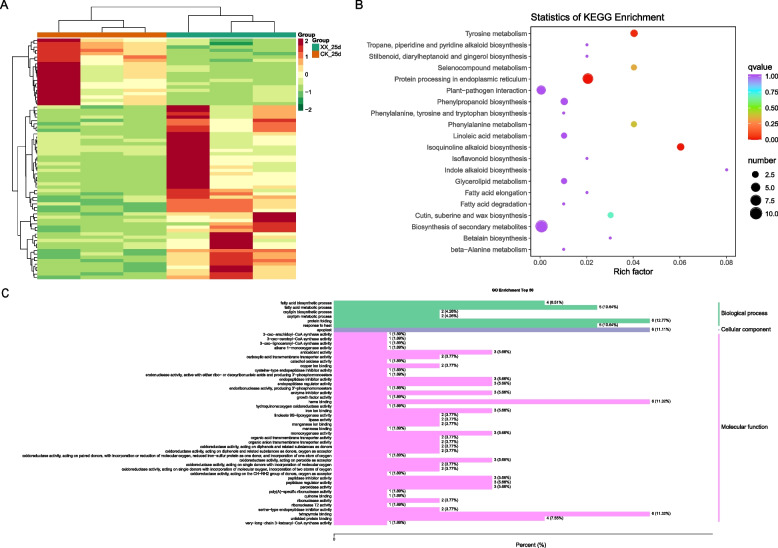
Differentially expressed genes (DEG) between the treatment and control groups at 25 d. **A** Heatmap representing the level of DEG across groups. **B** KEGG pathway enrichment analysis of the DEG identified in (A). **C** GO enrichment analysis of the DEG identified in (A). XX indicate uniconazole treatment, and CK indicate control

Differentially expressed genes (DEGs) between the treatment and control groups at 15 d were identified (Fig. 5A), which are involved in photosynthesis and oxidative phosphorylation pathways (Fig. 5B) and associated with photosynthesis, response to cytokinin, xylem development, and phenylpropanoid biosynthetic process (Fig. 5C). DEG identified at 25 d (Fig. 6A) were enriched in protein processing in the endoplasmic reticulum, tyrosine metabolism, and isoquinoline alkaloid biosynthesis pathways (Fig. 6B) and associated with tyrosine metabolic process, fatty acid biosynthetic process, oxylipin biosynthetic process, suberin biosynthetic process, lipid oxidation, phenylpropanoid metabolic process, and peroxidase activity (Fig. 6C). No common DEG were observed between 15 d and 25 d.

### Key pathways involved in uniconazole-induced dwarfism in banana

**Fig. 7:**
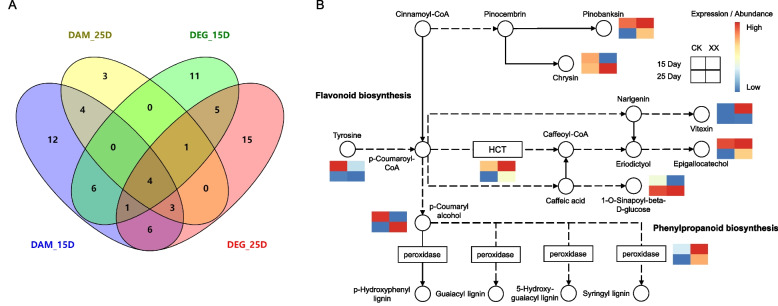
Key pathways involved in uniconazole-induced dwarfism. **A** Venn diagram showing the common enriched pathways for both DEG and DAM between 15 d and 25 d. **B** Schematic representation of the key pathways and the expression/abundance of genes and metabolites associated with phenylpropanoid and flavonoid biosynthesis. The top and bottom grids of each heatmap indicate the expression/abundance at 15 d and 25 d, respectively, and the left and right grids correspond to control and treatment groups, respectively. XX indicate uniconazole treatment, and CK indicate control

The common enriched pathways for both DEG and DAM between 15 d and 25 d were further examined, which included four key pathways: metabolic pathways, phenylpropanoid biosynthesis, flavonoid biosynthesis, and biosynthesis of secondary metabolites (Fig. 7A). Of these, the phenylpropanoid biosynthesis pathway comprises flavonoid biosynthesis. We further examined the differential factors in the phenylpropanoid biosynthesis pathway and found that the expression levels of shikimate O-hydroxycinnamoyltransferase (HCT) and peroxidase, and the abundance of pinobanksin, vitexin, and epigallocatechol increased in uniconazole-treated bananas compared with the control group (Fig. 7B). The quantitative real-time PCR (qRT-PCR) analysis also confirmed the overexpression of HCT and peroxidase genes in treatment group (Fig. S3).

## Discussion

Bananas treated with uniconazole showed a significant dwarf phenotype, indicating that the dwarfing induction was successful. The physiological and biochemical indexes of the treated group were also significantly different from those of the control group, and GA was significantly reduced in the treated group.

In previous studies, the biosynthesis of flavonoids and alkaloids has been reported to be activated in dwarfing plants, and tannins are associated with plant dwarfing. It has previously been reported that the biosynthesis or accumulation of flavonoids changes significantly in a variety of dwarf plants compared with the higher group. The expression levels of some flavonoid biosynthesis genes are significantly different in the dwarf Polish wheat mutant *Rht1* than in high Polish wheat. These changes increase flavonoid content [24]. *Rht1* gene encodes a GA signaling repressor that reduces the response to GA and limits the elongation of wheat stems [2, 25]. In *Seashore paspalum*, the dwarf phenotype of mutant *T51* is considered to be closely related to the upregulation of flavonoid biosynthesis in the phenylpropanoid pathway [26]. Over-accumulation of flavonoids has been reported not only in dwarf herbs, but also in woody apple dwarfing rootstocks [27]. Isoquinoline alkaloid biosynthesis was reported to be activated in the dwarf wheat infected with *Tilletia controversa* Kühn [28]. In our results, a variety of flavonoids were increased, and increased alkaloids were significantly enriched in isoquinoline alkaloids in the uniconazole-treated banana, similar to the changes previously reported in the dwarfing plants mentioned above. Various tannins have been reported to inhibit GA-induced plant growth [29, 30]. Although uniconazole was considered an inhibitor of GA biosynthesis in previous reports, we found no significant changes related to the GA biosynthesis pathway in bananas treated with uniconazole [9, 31]. The reported GA inhibitor tannin procyanidin B1 was increased in bananas treated with uniconazole, suggesting that reduced GA levels are not the only factor responsible for banana dwarfing, as the over-accumulation of tannins may inhibit GA activity and cause banana dwarfing [30]. We propose that increased levels of flavonoids, isoquinoline alkaloids, and tannins play important roles in uniconazole-induced dwarfing.

The phenylpropanoid pathway is involved in several physiological processes. In addition to flavonoid biosynthesis, the lignin synthesis pathway is involved in the phenylpropanoid pathway [32–35]. Previous studies have reported that the knockout or knockdown of one or more genes of the phenylpropanoid pathway can lead to dwarfism by reducing the lignin content [36]. Lignin is a complex, aromatic polymer mainly presenting in secondarily thickened cell walls and provides rigidity, strength, and hydrophobicity [37–39]. In some dwarf plants with enhanced flavonoid biosynthesis, lignin biosynthesis is downregulated. Compared with high Polish wheat, the expression levels of some lignin are significantly different in the dwarf Polish wheat mutants *Rht1*, leading to lignin level reduction [24]. In *S. paspalum*, the dwarf phenotype of mutant *T51* is considered to be closely related not only to the upregulation of flavonoid biosynthesis, but also to the downregulation of lignin biosynthesis [26]. In addition, delayed lignin accumulation was found in dwarfed transgenic rice expressing the α-L-arabinofuranosidase of Coprinopsis cinerea [40]. However, in some plants, lignin accumulation was also found to be detrimental to plant growth. Leaf, root and stem growth were significantly enhanced in transgenic aspen with the lignin biosynthetic pathway gene *Pt4CL1* downregulated [41]. In addition, banana plants overexpressed with VND1, VND2 or VND3 had increased lignin deposition. These transgenic banana plants showed stunted growth [42, 43]. Moreover, the reduction of lignin was observed in transgenic bananas overexpressing MusaNAC68 and was considered to be linked with the increase in the height of transgenic bananas [44]. Although peroxidase increased in uniconazole-treated bananas at both 15 d and 25 d, p-coumaryl alcohol, the substrate of peroxidase, only increased at 25 d and decreased at 15 d. At 25 d, peroxidase expression was lower than that at 15 d. The inconsistency in the time and degree of change of the substrate p-coumaryl alcohol and peroxidase could not provide sufficient evidence for the change in lignin biosynthesis. In addition, some other genes or metabolites in the phenylpropanoid synthesis pathway that are not involved in lignin biosynthesis also increased significantly under uniconazole treatment. P-Coumaroyl-CoA can be converted to p-coumaroyl alcohol or to the flavonoid epigallocatechol. HCT is an enzyme involved in the conversion of p-coumaroyl-CoA to epigallocatechol [45]. Both HCT and epigallocatechol increased in uniconazole-treated bananas at 15 d and 25 d. These results suggest that the enhanced biosynthesis of flavonoids may reduce the metabolic flux of lignin. The important plant hormone auxin and several auxin responsive factors (ARFs) have also been reported to regulate lignin synthesis, and *MYB* gene has previously been reported to be involved in auxin response and endothecium lignification of anther walls [46]. The substantial elevation of *MYB* transcription factors such as *MYB4a-like* and *MYB4b-like* factors was observed in transgenic bananas overexpressing MusaNAC68 [44]. However, no significant changes in the expression of *ARF* and *MYB* genes were identified in the uniconazole-treated bananas. The mechanism of dwarfism in uniconazole-treated bananas may be related to lignin biosynthesis during stem elongation, but the specific effect of lignin biosynthesis on banana growth need to be further studied.

In summary, in addition to a decrease in GA content, an increase in tannin procyanidin B1 content may contribute to banana dwarfing by inhibiting GA activity. Flavonoids and lignin are metabolites of the phenylpropanoid pathway, and there is a competitive relationship between them. The increase in flavonoid biosynthesis promoted the increased flow of metabolites towards flavonoid synthesis, which indirectly led to a decrease in lignin biosynthesis. Based on the above results, we propose a uniconazole-induced dwarfing mechanism hypothesis: over-accumulation of tannin inhibits the role of GA in banana growth, and abnormal lignin synthesis affects cell wall function, ultimately limiting cell expansion and causing dwarfism in uniconazole-treated bananas.

## Materials and methods

### Plant materials and treatment

Experiments were performed at the Libang Scientific Base of Guangxi Academy of Agricultural Sciences, located in Futang town in Wu Ming district, Nanning city, Guangxi province, China. The banana cultivar ‘Guijiao No.9’ was used, and the seeds were planted in January, 2021. We got the permission to collect Banana seeds. And the study protocol was complied with relevant institutional, national, and international guidelines and legislation. Uniconazole wettable powder (5%, Sichuan Runer Technology, China) was applied to the plants when they grew to 16–18 leaves. The powder was diluted to obtain different concentration gradients, and then 200 mL solutions were drenched along the base of the pseudostem, resulting a gradient dosage of 0.1 g/plant, 0.3 g/plant, and 0.5 g/plant. Plants treated with water at the same stage as described above were used as controls. Twenty plants were used for each uniconazole-treated and control group and used to measure the dwarfed phenotype. Leaves were collected at 15, 20, and 25 d after treatment for both the uniconazole-induced and control groups. A total of 100 g of leaf blade (without leaf vein) was clipped from the penultimate piece of leaf for each plant and immediately frozen in liquid nitrogen or stored in a refrigerator at − 80 °C. Three independent biological replicates were conducted for the subsequent measurement of physiological indices, cDNA library construction, and RNA sequencing for each uniconazole-treated and control group.

### Measurement of physiological indices

GA production was estimated using a tetramethyl benzidine (TMB) detection system after incubation with an HRP-conjugated antibody. GA content was quantified by measuring the absorbance at 450 nm. Other physiological indices of the leaf samples were assessed using a spectrophotometric method. Polyphenol oxidase (PPO) activity was assayed using pyrocatechol as the substrate. Superoxide dismutase (SOD) activity was measured using the xanthine oxidase method based on the production of O^2^ − •. Catalase (CAT) activity was examined by measuring H_2_O_2_ decomposition. Phenylalanine ammonia-lyase (PAL) activity was measured from the conversion of l-phenylalanine to trans-cinnamic acid. Soluble protein was quantified based on the reduction of Cu^2+^ to Cu^1+^ in an alkaline environment. PPO, SOD, CAT, PAL, and soluble protein activities were determined by measuring the absorbance at 410, 450, 240, 290, and 562 nm, respectively, and expressed as units/mg protein.

### Measurement of biochemical indices

The potassium, calcium, and magnesium contents were determined by atomic absorption spectroscopy (AAS, TAS-900 AFG, China). Total phosphorus content was measured using phosphorus molybdenum blue spectrophotometry at 660 nm, and total phosphorus was expressed as the concentrations of organic and inorganic phosphorus. Total nitrogen was determined by titration with ferrous ammonium sulfate using an azotometer. Si concentration was quantified using plasma atomic emission spectroscopy (ICP-AES).

### Metabolite extraction and LC-MS/MS analysis

Leaf samples were freeze-dried and crushed using a mixer mill (MM 400, Retsch). Lyophilized powder (100 mg) was dissolved in 1.2 mL of 70% methanol solution and kept at 4 °C overnight. After centrifugation at 12000 rpm for 10 min, the extracts were filtered (SCAA-104, 0.22 μm pore size; ANPEL, Shanghai, China). UPLC separation was performed using a 1.8 μm, 2.1 mm * 100 mm Agilent SB-C18 column. Linear ion trap (LIT) and triple quadrupole (QQQ) scans were acquired on an Applied Biosystems 4500 Q TRAP LC-MS/MS system, including an ESI Turbo ion–spray interface.

Metabolites were extracted and identified using the Metware database (Metware Biotechnology, Wuhan, China). VIP values for the identified metabolites were determined by OPLS-DA analysis using the R package MetaboAnalystR. Significantly regulated metabolites between groups were determined by VIP ≥ 1 and absolute log2FC (fold-change) ≥ 1 and were then subjected to metabolite set enrichment analysis (MSEA).

### RNA extraction and RNA-Seq analysis

Total RNA was extracted from leaf samples using the Qiagen RNeasy Plant Kit (Hilden, Germany), according to the manufacturer’s protocol. mRNAs were enriched by poly(A) selection from the extracted total RNA, and rRNA-depleted samples were prepared using the Illumina TruSeq RNA Sample Prep Kit to obtain a strand-specific library. Purification and size selection of cDNA were performed using AMPure XP beads, resulting in a median fragment size of 300 bp. The cDNA libraries were then checked using Qubit2.0, Agilent 2100, and sequenced using the Illumina Novaseq platform.

Raw data were preprocessed using fastp (v0.19.3) with parameters “–n_base_limit 15 –qualified_quality_phred 20,” and clean reads were then aligned to the banana reference genome (NCBI accession No. GCF_000313855.2) with HISAT2 (v2.1.0). Gene expression levels were quantified using featureCounts (v1.6.1), and fragments per kilobase of transcript per million fragments mapped (FPKM) was calculated. Pearson’s correlation coefficients between samples were computed, and principal component analysis (PCA) was performed based on gene expression levels. DESeq2 (v1.22.1) was used to perform differential gene expression analysis between groups. Genes with |log2foldchang| ≥ 1 and FDR < 0.05 were identified as significantly DEG. Gene ontology (GO) and Kyoto Encyclopedia of Genes and Genomes (KEGG) enrichment analyses were performed for DEG using clusterProfiler (v3.10.1).

### Quantitative real-time PCR (qRT-PCR) analysis

Primers for qRT-PCR were designed using Primer Premier software (5.0) and were synthesized commercially (TIANYI HUIYUAN, Wuhan, China). RNA was isolated using TRI Reagent Solution (Ambion, TR118), according to the manufacturer’s instructions. Reverse transcription was performed using HiScript QRT SuperMix for qPCR (Vazyme, Nanjing, China). The primers of HCT and peroxidase genes were listed in Table S4. β-actin was used as an internal control. Quantitative real-time PCR was subsequently performed using SYBR® Select Master Mix (CFX) on a StepOnePlusTM Real-Time System (Applied Biosystems). qPCR was performed using the ΔΔCt method.

### Statistical analysis

Pearson correlation coefficients (PCC) between samples were calculated using the cor function in R for both transcriptomic and metabolomic data. MSEA, GO, and KEGG enrichment analyses were performed using hypergeometric tests.

## Supplementary Information

**Additional file 1.**

**Additional file 2.**

## Data Availability

The sequencing data generated in the study are deposited to the NCBI SRA database under Bioproject No. PRJNA855237. The data will be released upon publication, and the link for reviewer is: https://dataview.ncbi.nlm.nih.gov/object/PRJNA855237?reviewer=ltgjsfssohfd875k887agpklgv

## Abbreviations

AAS: Atomic absorption spectroscopy
CAT: Catalase
DAM: Differentially abundant metabolite
DEG: Differentially expressed gene
FPKM: Fragments per kilobase of transcript per million fragments mapped
GA: Gibberellin
GO: Gene ontology
HCT: O-hydroxycinnamoyltransferase
ICP-AES: Plasma atomic emission spectroscopy
KEGG: Kyoto Encyclopedia of Genes ad Genome
MS: Mass spectrometry
PAL: Phenylalanine ammonia-lyase
PCA: Principal component analysis
PCC: Pearson correlation coefficients
PPO: Polyphenol oxidas
qRT-PCR: Quantitative real-time PCR
SOD: Superoxide dismutas
TMB: Tetramethylbenzidine
UPLC: Ultraperformance liquid chromatography
VIP: Variable importance in projection
