# Supplementary material for: Integrated metabolomic and transcriptomic analyses of regulatory mechanisms associated with uniconazole-induced dwarfism in banana
Source: BMC Plant Biol. 2022 Dec 28;22:614. doi: 10.1186/s12870-022-04005-w (PMC9795754; doi:10.1186/s12870-022-04005-w)
Supplement: Supplementary file 1 — Additional file 1. [file 12870_2022_4005_MOESM1_ESM.pptx]

## Slide 1
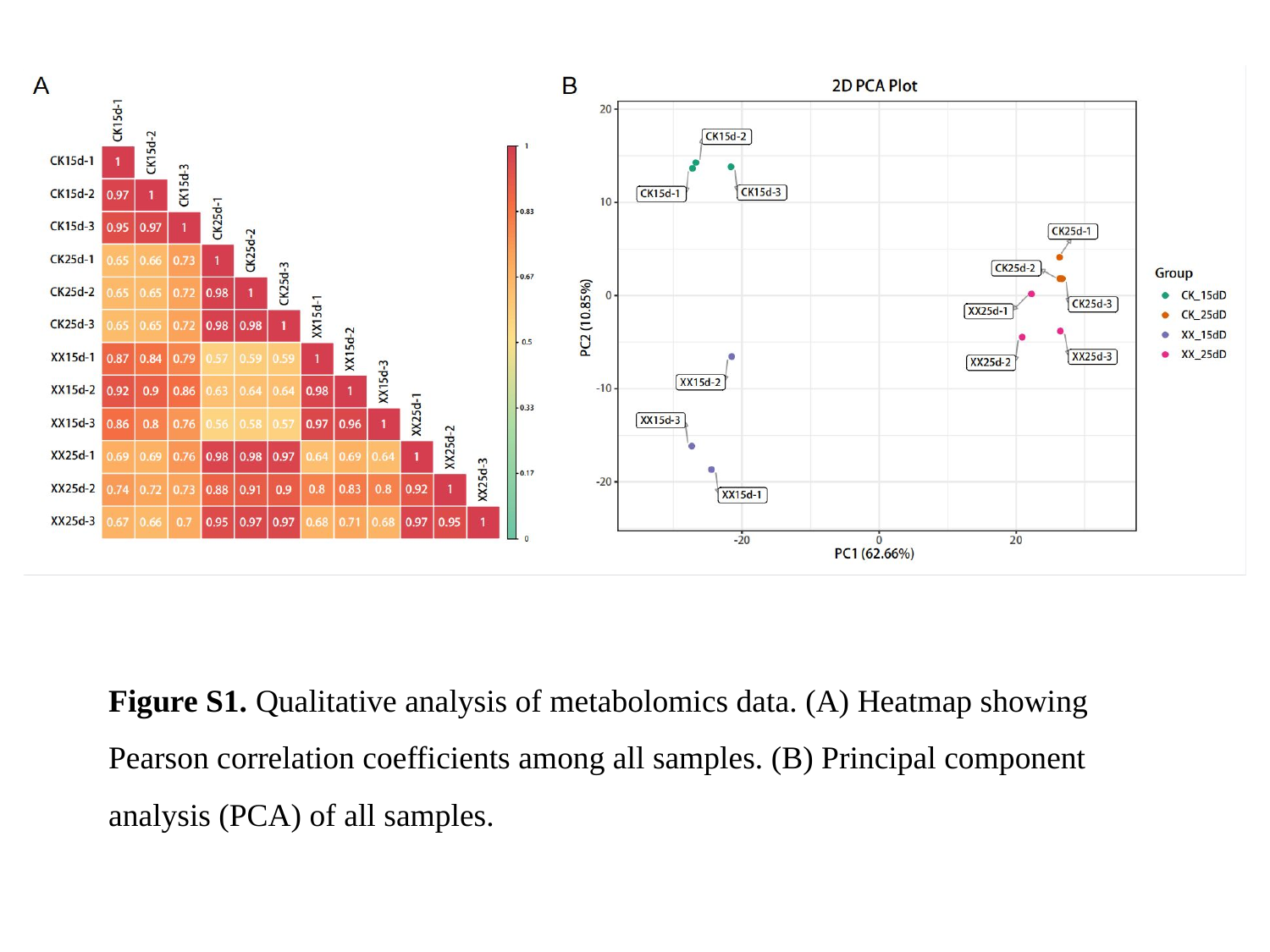

Figure S1. Qualitative analysis of metabolomics data. (A) Heatmap showing Pearson correlation coefficients among all samples. (B) Principal component analysis (PCA) of all samples.

## Slide 2
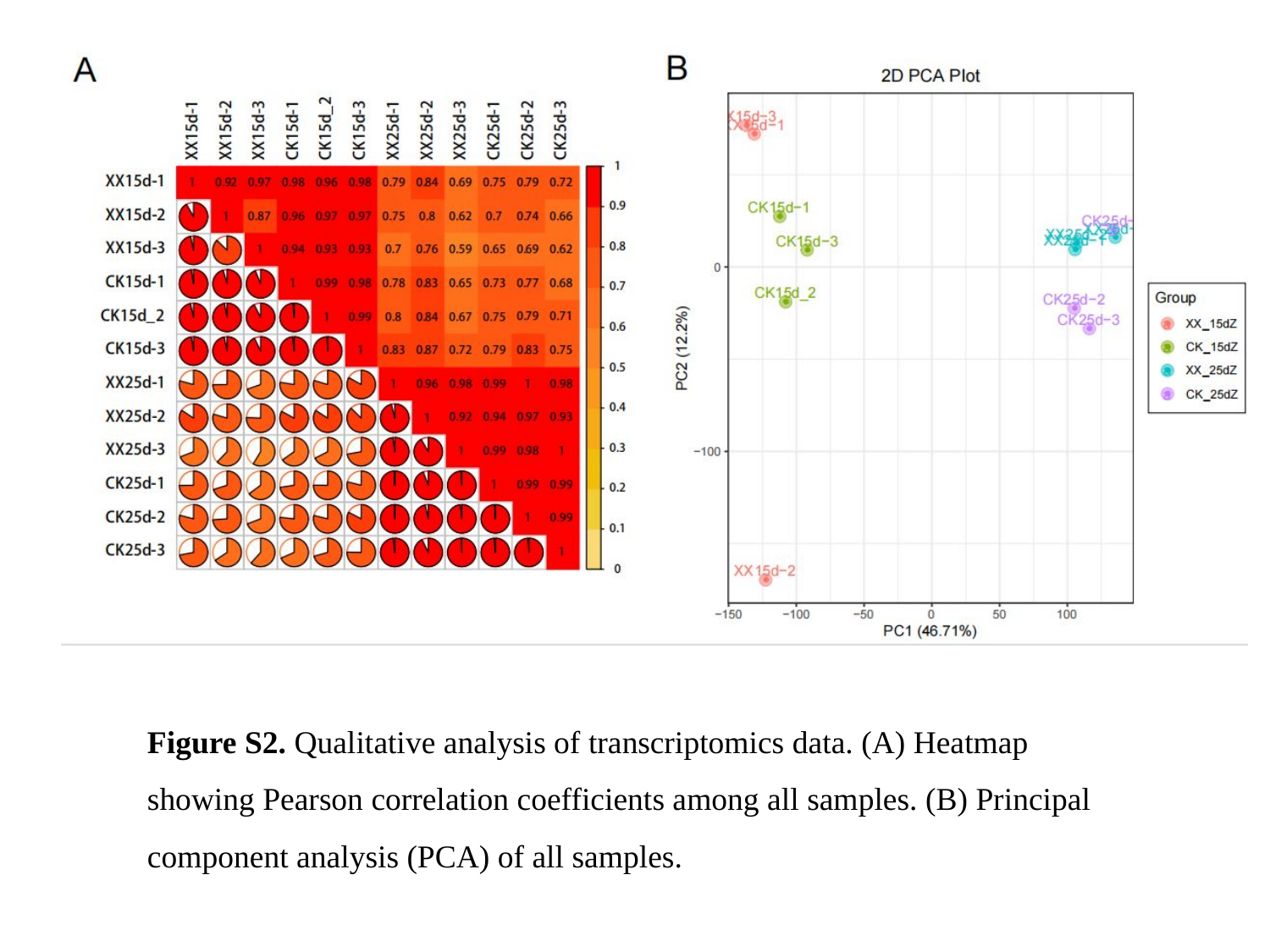

Figure S2. Qualitative analysis of transcriptomics data. (A) Heatmap showing Pearson correlation coefficients among all samples. (B) Principal component analysis (PCA) of all samples.

## Slide 3
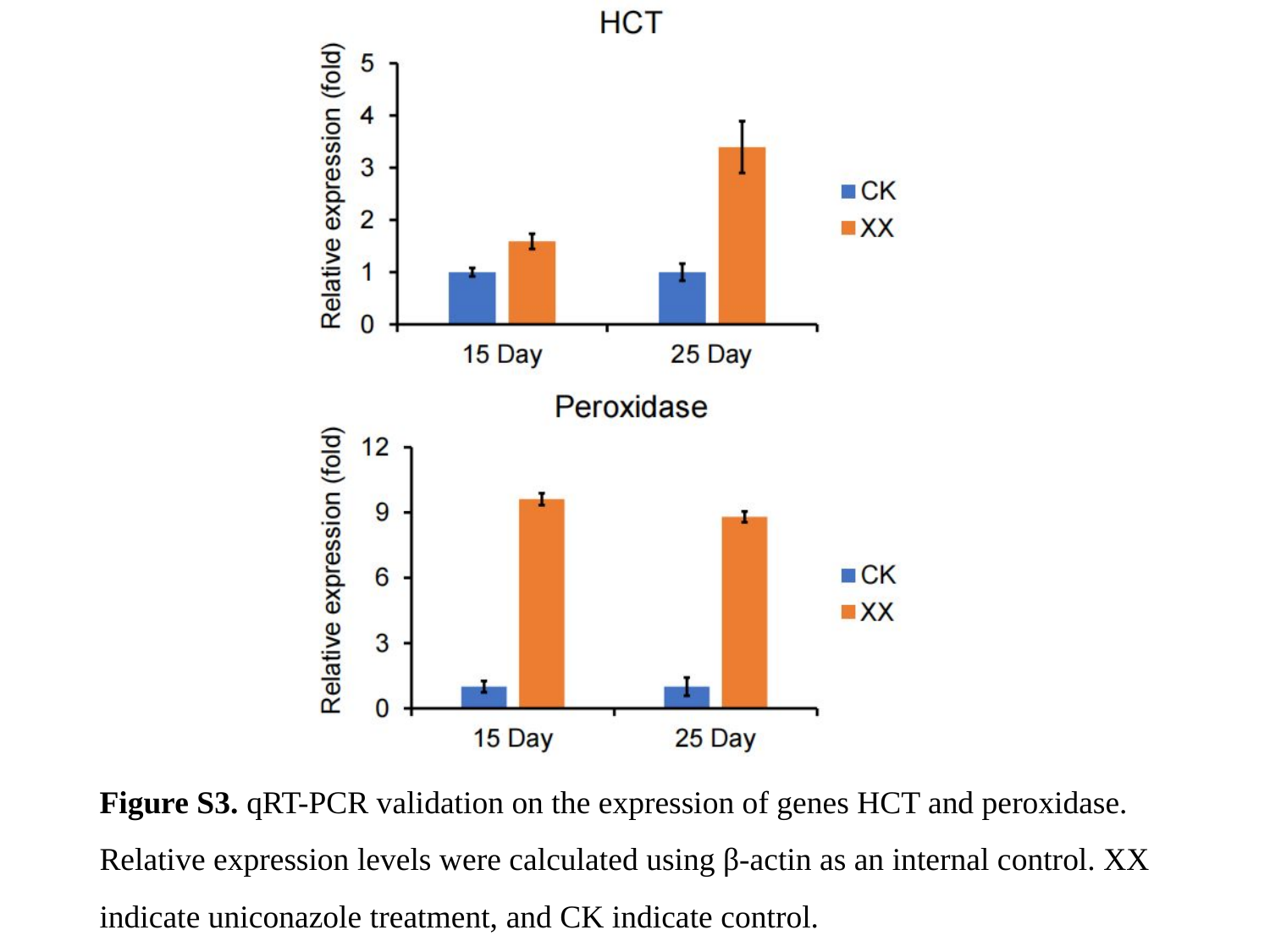

Figure S3. qRT-PCR validation on the expression of genes HCT and peroxidase. Relative expression levels were calculated using β-actin as an internal control. XX indicate uniconazole treatment, and CK indicate control.
